# Supplementary figures and images for: Association of vaginal IL-4, IL-6, IL-8, IL-17, IFN-γ, and dietary intake with IBD status and vaginal microbiota in pregnant individuals
Source: PLoS One. 2026 Jan 14;21(1):e0335178. doi: 10.1371/journal.pone.0335178 (PMC12803450; doi:10.1371/journal.pone.0335178)

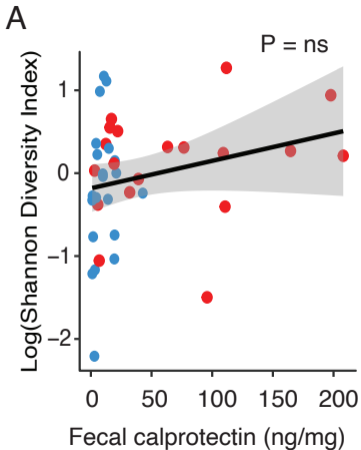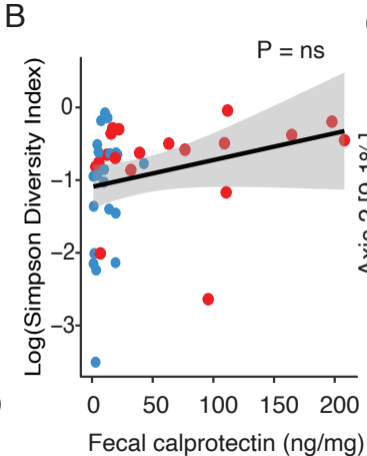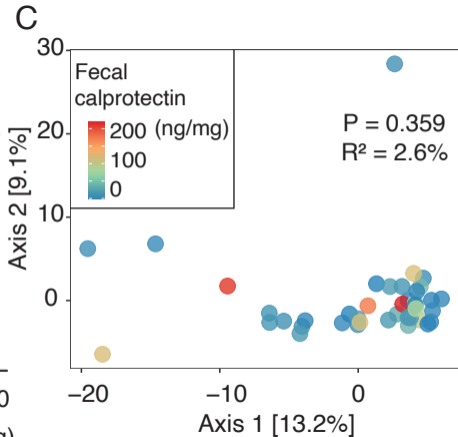

Supplement: S1 Fig — (A, B) Correlation of fecal calprotectin levels with vaginal microbiota alpha diversity indexes, Shannon (A) and Simpson (B). “ns” indicates that fecal calprotectin was not retained in the final linear model. (C) Beta diversity for vaginal microbiota using PCoA based on Aitchison distances colored by fecal calprotectin levels (ng/mg). (PDF) [file pone.0335178.s003.pdf]

A

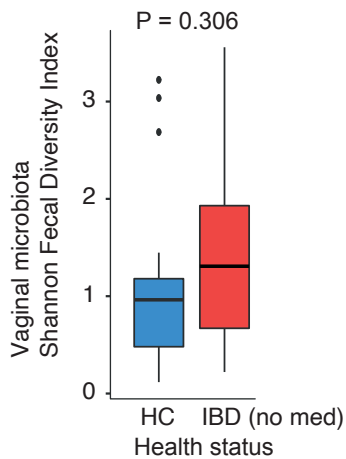

B

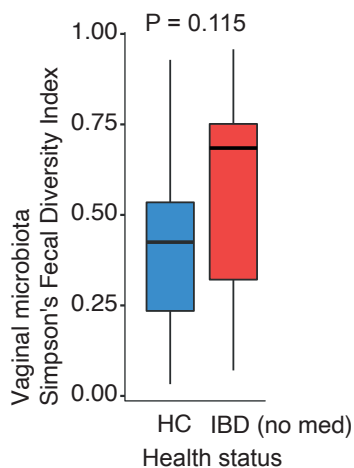

C

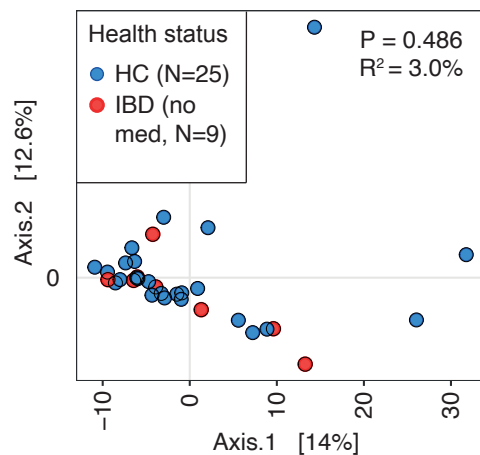

D

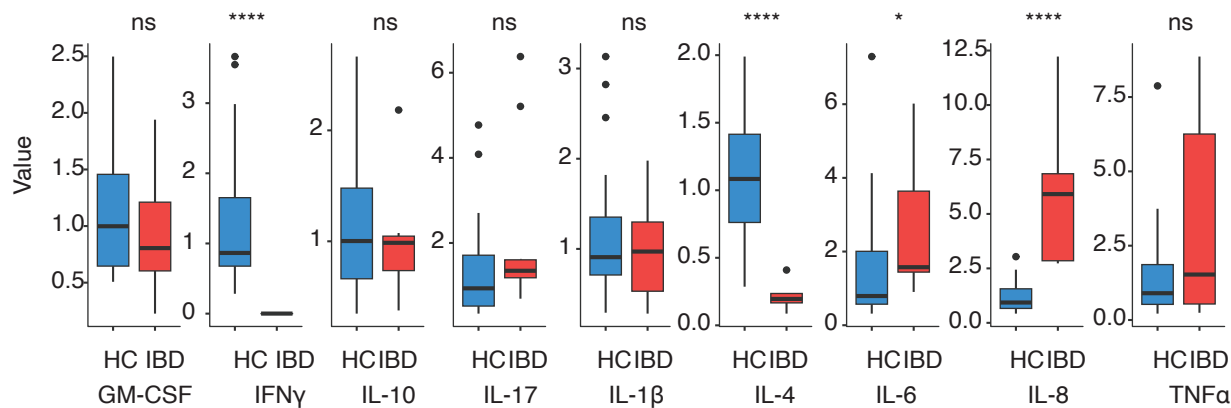

Supplement: S2 Fig — (A, B) Comparison of vaginal microbiota alpha diversity indexes, Shannon (A) and Simpson (B), between HC and IBD patients not receiving medication. (C) Beta diversity visualization of vaginal microbiota using PCoA based on Aitchison distances, colored by health status. (D) Relative fold changes of vaginal cytokine gene expression normalized to GAPDH in HC and IBD groups (no medication). Boxplots show median values and interquartile ranges. * p < 0.05, ****p < 0.0005. (PDF) [file pone.0335178.s004.pdf]
