# Supplementary material for: Association of vaginal IL-4, IL-6, IL-8, IL-17, IFN-γ, and dietary intake with IBD status and vaginal microbiota in pregnant individuals
Source: PLoS One. 2026 Jan 14;21(1):e0335178. doi: 10.1371/journal.pone.0335178 (PMC12803450; doi:10.1371/journal.pone.0335178)
Supplement: S1 Table — Details the total number of sequences and amplicon sequence variants (ASVs) in 16S vaginal samples. (PDF) [file pone.0335178.s005.pdf]

S1 Table. Overview of Sequence and Variants Counts for *16SrRNA* Sequencing of Vaginal Samples. Details the total number of sequences and amplicon sequence variants (ASVs) by *16SrRNA* sequencing of vaginal samples.

	**Vaginal samples**		
**Parameter**	**Raw sequences**	**Post-filtering (<0.1%)**	**Post-filtering/rarefaction ***
N samples	48	48	48
Total number of seqs	2,751,605	2,742,919	336,000
Mean seqs/sample	57,325	57,144	7,000
Standard deviation of seqs/sample	24,010	23,945	N/A
Max number of seqs in a sample	123,825	123,720	7,000
Min number of seqs in a sample	12,916	12,872	7,000
Total number of different ASVs or features	1,425	573	563
Mean ASV or features/sample	48	36	33
Standard deviation of ASV or features/sample	38	23	23
Max number of ASVs or features in a sample	208	109	105
Min number of ASVs or features in a sample	6	6	5

* Sequences were rarefied at 7,000 sequences/sample, representing the highest number of sequences that included all vaginal samples
