# Supplementary material for: Association of vaginal IL-4, IL-6, IL-8, IL-17, IFN-γ, and dietary intake with IBD status and vaginal microbiota in pregnant individuals
Source: PLoS One. 2026 Jan 14;21(1):e0335178. doi: 10.1371/journal.pone.0335178 (PMC12803450; doi:10.1371/journal.pone.0335178)
Supplement: S2 Table — List of primers (forward and reverse) targeting cytokine genes for qRT-PCR analysis, with sequences displayed in the 5’ to 3’ orientation. (PDF) [file pone.0335178.s006.pdf]

S2 Table. Oligonucleotide sequences used for determining vaginal cytokine gene expression. This table provides a list of primers (forward and reverse) targeting cytokine genes for qRT-PCR analysis, with sequences displayed in the 5’ to 3’ orientation.

Serial No	Target gene (qRT primer)	Sequence (5’ - 3’)
1	IL-1 beta (F)	CAAGAGTGCTGGAGCGATAA
2	IL-1 beta (R)	CCGTTACAGCGGAGAGATATAAG
3	TNFa(F)	CACAGAAGACACTCAGGGAAAG
4	TNFa (R)	TCGAGGGAGTCACCCTTAAA
5	IL-6 (F)	GGAGGAGCCAAAGCTCAATAA
6	IL-6 (R)	ACTCCACACCACAAGAAGATG
7	IL-4 (F)	CCTGCTTCATGAGGGAAACT
8	IL-4 (R)	GGTGACAGAACAAGACCCTATC
9	IL-8 (F)	GAAAGGAAGTAGCTGGCAGAG
10	IL-8 (R)	GGGTGGAAAGGTTTGGAGTAT
11	IL-10 (F)	TGGAGTGAGTCCTGGAGAAATA
12	IL-10 (R)	CTCCATGTCCATCACACTTAGG
13	IFN-γ (F)	CCAGCCATCCTCAGAAATGT
14	IFN-γ (R)	CTTGCATCTCCTCACTCTAACC
15	IL-17 (F)	CCATAGTGAAGGCAGGAATCA
16	IL-17 (R)	GAGGTGGATCGGTTGTAGTAATC
17	GM-CSF (F)	CCTCCAGCAGGAATGTCTTAAT
18	GM-CSF (R)	TCTGACTCCTTGGGATGAAATG
19	GAPDH (F)	GTGCTCCCACTCCTGATTT
20	GAPDH (R)	CCTTCTCTAAGTCCCTCCTACA
