# Supplementary material for: Association of vaginal IL-4, IL-6, IL-8, IL-17, IFN-γ, and dietary intake with IBD status and vaginal microbiota in pregnant individuals
Source: PLoS One. 2026 Jan 14;21(1):e0335178. doi: 10.1371/journal.pone.0335178 (PMC12803450; doi:10.1371/journal.pone.0335178)
Supplement: S3 Table — Demographic and clinical characteristics of pregnant individuals with CD or UC recruited for the study between 2019 and 2022. (PDF) [file pone.0335178.s007.pdf]

S3 Table. Demographic and clinical variables for pregnant individuals with Crohn's Disease (CD) or Ulcerative Colitis (UC). Demographic and clinical characteristics of pregnant individuals with CD or UC recruited for the study between 2019 and 2022.

Demographics and clinical variables	CD (N=18)	UC (N=5)	Overall (N=23)	P value*
**Age**				*0.822*
Mean (SD)	33.2 (4.86)	33.4 (4.22)	33.3 (4.63)	
Median [Min, Max]	33.5 [22.0, 41.0]	32.0 [28.0, 39.0]	33.0 [22.0, 41.0]	
**BMI categories ****				*0.126*
Underweight	1 (5.6%)	0 (0%)	1 (4.3%)	
Normal	10 (55.6%)	2 (40.0%)	12 (52.2%)	
Overweight	6 (33.3%)	2 (40.0%)	8 (34.8%)	
Obese	1 (5.6%)	1 (20.0%)	2 (8.7%)	
**Race**				-
White	18 (100%)	5 (100%)	23 (100%)	
Asian	0 (0%)	0 (0%)	0 (0%)	
Other	0 (0%)	0 (0%)	0 (0%)	
**Gestational diabetes**				*0.053*
Yes	1 (5.6%)	2 (40.0%)	3 (13.0%)	
No	14 (77.8%)	2 (40.0%)	16 (69.6%)	
Information unavailable	3 (16.7%)	1 (20.0%)	4 (17.4%)	
**Use of antibiotic during pregnancy**				*1.000*
No	15 (83.3%)	4 (80.0%)	19 (82.6%)	
Yes	3 (16.7%)	0 (0%)	3 (13.0%)	
Information unavailable	0 (0%)	1 (20.0%)	1 (4.3%)	
**Disease activity *****				*0.456*
Mild disease	5 (27.8%)	1 (20.0%)	6 (26.1%)	
Remission	10 (55.6%)	3 (60.0%)	13 (56.5%)	
Information unavailable	3 (16.7%)	1 (20.0%)	4 (17.4%)	
**Use of IBD medication**				*0.342*
No	6 (33.3%)	3 (60.0%)	9 (39.1%)	
Yes	12 (66.7%)	2 (40.0%)	14 (60.9%)	
**Preterm**				*0.272*
No	12 (66.7%)	3 (60.0%)	15 (65.2%)	
Yes	2 (11.1%)	2 (40.0%)	4 (17.4%)	
Missing	4 (22.2%)	0 (0%)	4 (17.4%)	
**Infant birth weight (g)**				*0.411*
Mean (SD)	3100 (515)	3330 (169)	3150 (470)	
Median [Min, Max]	3230 [1810, 3710]	3230 [3180, 3540]	3230 [1810, 3710]	
**Fecal calprotectin (ng/mg)**				*1.000*
Mean (SD)	69.4 (65.9)	59.5 (42.7)	67.3 (60.9)	
Median [Min, Max]	45.2 [3.11, 208]	45.2 [6.89, 111]	45.2 [3.11, 208]	
**Community State Types (CSTs)**				*0.209*
I	7 (38.9%)	2 (40.0%)	9 (39.1%)	
II	4 (22.2%)	0 (0%)	4 (17.4%)	
III	2 (11.1%)	3 (60.0%)	5 (21.7%)	
IV-C	3 (16.7%)	0 (0%)	3 (13.0%)	
V	2 (11.1%)	0 (0%)	2 (8.7%)	

*Fisher's exact test for categorical variables and Wilcoxon test for continuous variables.

**BMIs categories correspond to the WHO’s classifications: Underweight (<18.5), normal weight (18.5–24.9), overweight (≥25.0), and obese (≥30).

***Disease activity was estimated using the Harvey Bradshaw Index and the Mayo score for individuals with Crohn's Disease or Ulcerative colitis, respectively.
