# Supplementary material for: Association of vaginal IL-4, IL-6, IL-8, IL-17, IFN-γ, and dietary intake with IBD status and vaginal microbiota in pregnant individuals
Source: PLoS One. 2026 Jan 14;21(1):e0335178. doi: 10.1371/journal.pone.0335178 (PMC12803450; doi:10.1371/journal.pone.0335178)
Supplement: S4 Table — Distribution of CSTs across different health statuses, and mean and median or fecal calprotectin levels, and cytokine expression levels in vaginal samples. CST I is dominated by Lactobacillus crispatus, CST II by L. gasseri, CST III by L. iners, CST IV-C by a diverse set of anaerobes, and CST V by L. jensenii. (PDF) [file pone.0335178.s008.pdf]

S4 Table. Distribution of vaginal microbial Community State Types (CSTs) by health status, fecal calprotectin levels, and vaginal cytokine expression. Distribution of CSTs across different health statuses, and mean and median or fecal calprotectin levels, and cytokine expression levels in vaginal samples. CST I is dominated by *Lactobacillus crispatus*, CST II by *L. gasseri*, CST III by *L. iners*, CST IV-C by a diverse set of anaerobes, and CST V by *L. jensenii*.

	Community State Types							
Variables	I (N=21)	II (N=7)	III (N=14)	IV-C (N=4)	V (N=2)	Overall (N=48)	P value*	Signif
**Health status**							***0.369***	ns
HC	12 (57.1%)	3 (42.9%)	9 (64.3%)	1 (25.0%)	0 (0%)	25 (52.1%)		
IBD	9 (42.9%)	4 (57.1%)	5 (35.7%)	3 (75.0%)	2 (100%)	23 (47.9%)		
**Fecal calprotectin (ng/mg)**							***0.374***	ns
Mean (SD)	41.5 (62.9)	36.6 (47.2)	26.3 (26.5)	40.4 (47.8)	120 (124)	39.3 (53.4)		
Median [Min, Max]	9.86 [1.20, 198]	12.4 [1.86, 109]	19.4 [1.12, 95.8	19.5 [10.7, 112]	120 [32.0, 208]	15.7 [1.12, 208]		
Missing	5 (23.8%)	0 (0%)	0 (0%)	0 (0%)	0 (0%)	5 (10.4%)		
**IL-6**							***0.114***	ns
Mean (SD)	3.8 (4.1)	4.4 (3.4)	2.9 (2.3)	7.5 (3.9)	8.7 (0.05)	4.1 (3.7)		
Median	2.14	4.05	2.5	7.2	8.76	3.12		
**IL-4**							***0.474***	ns
Mean (SD)	0.761 (0.658)	0.850 (0.762)	0.855 (0.684)	0.377 (0.301)	0.193 (0.140)	0.746 (0.651)		
Median	0.421	0.877	0.581	0.277	0.193	0.428		
**IL-1**							***0.086***	ns
Mean (SD)	1.22 (0.453)	1.18 (0.403)	1.04 (0.392)	0.763 (0.519)	2.01 (0.321)	1.16 (0.469)		
Median	1.23	1.06	1.02	0.655	2.01	1.06		
**TNF-alpha**							***0.237***	ns
Mean (SD)	3.46 (3.14)	4.42 (4.05)	3.00 (2.51)	2.48 (1.90)	11.2 (1.79)	3.71 (3.33)		
Median	1.32	4.74	1.69	1.93	11.2	2.1		
**IFN-gamma**							***0.431***	ns
Mean (SD)	0.583 (0.671)	0.663 (0.951)	0.776 (0.751)	0.152 (0.303)	0.00340 (0.00157	0.591 (0.715)		
Median	0.561	0.001	0.712	0.000763	0.0034	0.511		
**IL-8**							***0.201***	ns
Mean (SD)	3.05 (3.05)	3.96 (4.26)	2.25 (2.12)	4.15 (4.24)	11.5 (2.62)	3.39 (3.48)		
Median	1.57	1.26	1.79	2.68	11.5	1.93		
**GM-CSF**							***0.076***	ns
Mean (SD)	1.04 (0.453)	1.10 (0.561)	1.15 (0.488)	0.689 (0.565)	4.31 (0.303)	1.19 (0.815)		
Median	1.02	0.917	1.08	0.485	4.31	1.02		
**IL-10**							***0.176***	ns
Mean (SD)	1.15 (0.625)	1.27 (0.745)	1.20 (0.496)	1.09 (0.500)	5.59 (0.473)	1.36 (1.06)		
Median	1	1.17	1.11	0.922	5.59	1.09		
**IL-17**							***0.096***	ns
Mean (SD)	1.93 (1.20)	2.60 (1.31)	1.59 (1.08)	1.85 (1.26)	6.03 (1.09)	2.09 (1.44)		
Median	2.07	2.49	1.17	2.08	6.03	1.99		

* Fisher's exact test for categorical variables and Kruskal-Wallis test for continuous variables
