# Supplementary material for: Association of vaginal IL-4, IL-6, IL-8, IL-17, IFN-γ, and dietary intake with IBD status and vaginal microbiota in pregnant individuals
Source: PLoS One. 2026 Jan 14;21(1):e0335178. doi: 10.1371/journal.pone.0335178 (PMC12803450; doi:10.1371/journal.pone.0335178)
Supplement: S5 Table — HEI-2015 and individual dietary component scores for individuals with IBD and HC. (PDF) [file pone.0335178.s009.pdf]

S5 Table. HEI-2015 scores and dietary components by health status. HEI-2015 and individual dietary component scores for individuals with IBD and HC.

HEI-2015 dietary components (scoring scale)	IBD<br>(N=22)	HC<br>(N=25)	Overall<br>(N=47)	P value*
**Healthy Eating Index (0-100)**				*0.175*
Mean (SD)	66.6 (13.8)	61.4 (13.2)	63.8 (13.6)	
Median [Min, Max]	67.5 [45.0, 91.0]	64.0 [35.0, 84.0]	65.0 [35.0, 91.0]	
**Total vegetable (0-5)**				*0.361*
Mean (SD)	3.68 (1.39)	4.20 (1.12)	3.96 (1.27)	
Median [Min, Max]	4.00 [1.00, 5.00]	5.00 [1.00, 5.00]	5.00 [1.00, 5.00]	
**Greens and beans (0-5)**				*0.179*
Mean (SD)	4.41 (1.47)	3.48 (1.90)	3.91 (1.75)	
Median [Min, Max]	5.00 [0, 5.00]	5.00 [0, 5.00]	5.00 [0, 5.00]	
**Total fruit (0-5)**				*0.279*
Mean (SD)	3.23 (1.57)	2.80 (1.58)	3.00 (1.57)	
Median [Min, Max]	3.50 [0, 5.00]	3.00 [0, 5.00]	3.00 [0, 5.00]	
**Whole fruit (0-5)**				*0.281*
Mean (SD)	3.82 (1.76)	3.44 (1.87)	3.62 (1.81)	
Median [Min, Max]	5.00 [0, 5.00]	5.00 [0, 5.00]	5.00 [0, 5.00]	
**Whole grains (0-10)**				*0.728*
Mean (SD)	5.36 (3.27)	5.52 (3.27)	5.45 (3.24)	
Median [Min, Max]	5.50 [0, 10.0]	6.00 [0, 10.0]	6.00 [0, 10.0]	
**Dairy (0-10)**				*0.659*
Mean (SD)	6.68 (2.23)	6.76 (2.60)	6.72 (2.41)	
Median [Min, Max]	6.00 [3.00, 10.0]	7.00 [3.00, 10.0]	6.00 [3.00, 10.0]	
**Total protein foods (0-5)**				*0.508*
Mean (SD)	4.64 (0.727)	4.32 (1.03)	4.47 (0.905)	
Median [Min, Max]	5.00 [3.00, 5.00]	5.00 [1.00, 5.00]	5.00 [1.00, 5.00]	
**Seafood and plant protein (0-5)**				*0.212*
Mean (SD)	3.86 (1.83)	3.36 (1.78)	3.60 (1.80)	
Median [Min, Max]	5.00 [0, 5.00]	3.00 [0, 5.00]	5.00 [0, 5.00]	
**Fatty acid ratio (0-10)**				*0.162*
Mean (SD)	5.50 (3.60)	3.04 (2.94)	4.19 (3.46)	
Median [Min, Max]	5.00 [0, 10.0]	2.00 [0, 10.0]	3.00 [0, 10.0]	
**Sodium (0-10)**				*0.217*
Mean (SD)	5.84 (2.79)	5.82 (3.22)	5.83 (2.97)	
Median [Min, Max]	6.00 [1.00, 10.0]	6.50 [0, 10.0]	6.00 [0, 10.0]	
**Refined grains (0-10)**				*0.251*
Mean (SD)	6.09 (4.10)	6.76 (2.79)	6.45 (3.44)	
Median [Min, Max]	8.00 [0, 10.0]	7.00 [0, 10.0]	8.00 [0, 10.0]	
**Added sugar (0-10)**				*0.514*
Mean (SD)	8.18 (1.79)	8.20 (1.91)	8.19 (1.84)	
Median [Min, Max]	9.00 [5.00, 10.0]	9.00 [5.00, 10.0]	9.00 [5.00, 10.0]	
**Saturated fats (0-10)**				*0.847*
Mean (SD)	5.23 (3.32)	3.48 (3.00)	4.30 (3.24)	
Median [Min, Max]	5.50 [0, 10.0]	3.00 [0, 10.0]	4.00 [0, 10.0]	

* Wilcoxon test
