# Supplementary material for: Association of vaginal IL-4, IL-6, IL-8, IL-17, IFN-γ, and dietary intake with IBD status and vaginal microbiota in pregnant individuals
Source: PLoS One. 2026 Jan 14;21(1):e0335178. doi: 10.1371/journal.pone.0335178 (PMC12803450; doi:10.1371/journal.pone.0335178)
Supplement: S6 Table — HEI-2015 scores and individual dietary component distributions among vaginal CSTs. CST I is dominated by L. crispatus, CST II by L. gasseri, CST III by L. iners, CST IV-C by diverse anaerobes, and CST V by L. jensenii. (PDF) [file pone.0335178.s010.pdf]

S6 Table. HEI-2015 score and individual dietary components scoring distribution by Community State Types (CSTs).

Diet variables	Community State Types I (N=21)	II (N=6)	III (N=14)	IV-C (N=4)	V (N=2)	Overall (N=47)	P value*
**Healthy Eating Index (total score 0-100)**							*0.035*
Mean (SD)	**68.4 (11.3)**	61.0 (15.7)	**55.2 (13.7)**	69.0 (9.49)	73.5 (13.4)	63.8 (13.6)	
Median	**67**	65.5	**53**	65.5	73.5	65	
**Total vegetables (0-5)**							*0.007*
Mean (SD)	**4.57 (0.978)**	3.50 (1.38)	**3.21 (1.12)**	4.25 (1.50)	3.50 (2.12)	3.96 (1.27)	
Median	**5**	4	**3**	5	3.5	5	
**Greens and Beans (0-5)**							*0.169*
Mean (SD)	4.38 (1.36)	3.50 (2.07)	3.07 (2.20)	4.75 (0.500)	4.50 (0.707)	3.91 (1.75)	
Median	5	4.5	4	5	4.5	5	
**Total fruit (0-5)**							*0.016*
Mean (SD)	2.86 (1.31)	3.33 (1.86)	2.29 (1.59)	4.75 (0.500)	5.00 (0)	3.00 (1.57)	
Median	3	3.5	2	5	5	3	
**Whole fruit (0-5)**							*0.271*
Mean (SD)	3.81 (1.72)	3.83 (1.60)	2.79 (2.12)	4.50 (1.00)	5.00 (0)	3.62 (1.81)	
Median	5	4.5	3	5	5	5	
**Whole grains (0-10)**							*0.947*
Mean (SD)	5.52 (3.33)	5.33 (3.14)	5.14 (3.35)	5.50 (4.43)	7.00 (0)	5.45 (3.24)	
Median	6	5.5	4.5	6	7	6	
**Dairy (0-10)**							*0.919*
Mean (SD)	6.62 (2.52)	6.00 (2.45)	6.93 (2.53)	7.25 (1.71)	7.50 (3.54)	6.72 (2.41)	
Median	6	6	6.5	7.5	7.5	6	
**Total protein foods (0-5)**							*0.482*
Mean (SD)	4.33 (1.11)	5.00 (0)	4.50 (0.760)	4.50 (0.577)	4.00 (1.41)	4.47 (0.905)	
Median	5	5	5	4.5	4	5	
**Seafood and plant protein (0-5)**							*0.112*
Mean (SD)	4.24 (1.37)	3.67 (2.16)	2.79 (1.89)	2.50 (2.38)	4.50 (0.707)	3.60 (1.80)	
Median	5	5	2	2.5	4.5	5	
**Fatty acid ratio (0-10)**							*0.306*
Mean (SD)	4.95 (3.34)	2.67 (3.27)	3.36 (3.52)	5.25 (3.20)	4.50 (6.36)	4.19 (3.46)	
Median	4	1.5	2	4	4.5	3	
**Sodium (0-10)**							*0.305*
Mean (SD)	6.57 (2.60)	6.00 (3.35)	4.57 (3.27)	5.00 (2.94)	8.00 (1.41)	5.83 (2.97)	
Median	7	7	3.5	5.5	8	6	
**Refined grains (0-10)**							*0.627*
Mean (SD)	6.62 (3.80)	6.83 (2.86)	5.36 (3.50)	7.50 (2.52)	9.00 (1.41)	6.45 (3.44)	
Median	8	6.5	5	8	9	8	
**Added sugar (0-10)**							*0.020*
Mean (SD)	**9.10 (1.51)**	8.00 (1.79)	**7.07 (1.82)**	7.75 (2.06)	8.00 (1.41)	8.19 (1.84)	
Median	**10**	8.5	**6.5**	7.5	8	9	
**Saturated fats (0-10)**							*0.532*
Mean (SD)	5.10 (3.46)	2.67 (2.50)	3.79 (3.04)	4.75 (3.30)	3.50 (4.95)	4.30 (3.24)	
Median	5	2.5	3.5	6	3.5	4	

* Kruskal-Wallis test followed by Wilcoxon tests for significant variables; bolded pairs indicate significance for pairwise comparisons adjusted using False Discovery Rate (FDR) methods to control for multiple comparisons (adjusted P <
