# Supplementary material for: Association of vaginal IL-4, IL-6, IL-8, IL-17, IFN-γ, and dietary intake with IBD status and vaginal microbiota in pregnant individuals
Source: PLoS One. 2026 Jan 14;21(1):e0335178. doi: 10.1371/journal.pone.0335178 (PMC12803450; doi:10.1371/journal.pone.0335178)
Supplement: Attachment — Submitted filename: peer review 2.docx [file pone.0335178.s011.docx]

Title: Association of vaginal inflammatory markers and diet with the vaginal microbiota of pregnant individuals with or without IBD

I really enjoyed reading this article. It is well written and follows a nice sequence of experiments to help understand the research conducted for this paper.

**Summary of the paper:** This study used samples from MELODY trial to study the vaginal microbiota of pregnant women with or without IBD. They analysed the samples to identify the immune profile of vaginal mucosa. The microbiota diversity and CST composition was similar for IBD and healthy individuals. However, the authors identified elevated cytokines such as IL-6, IL-8 which are associated with poorer pregnancy outcomes and lower levels of IL-4 and IFN-g. Thereafter they also studied the impact of diet – especially added sugars and dairy intake on vaginal microbiota diversity and found lower added sugars and lower dairy intake was associated with lower microbiota diversity. Interestingly lower added sugar was associated with higher levels of *L. crispatus* which is a beneficial microbe. Finally, they also showed association between diet and inflammatory cytokines with total vegetables and whole grain being negatively associated with expression of IFN-γ and IL-8 in pregnant women with IBD.

**Review comments:**

**Title:** Recommend to add IL-6, IL-8 and IFN-g to title (e.g. association of inflammatory markers such as IL-6…..and diet with the vaginal……) to improve impact.

**Introduction:**

**General:** Overall, this section needs to be strengthened. The introduction is not supported by sufficient data and the authors need to generate reader interest in the topic by connecting bacterial vaginosis, IBD and pregnancy outcomes. Also, there are few articles available which talk about inflammatory markers in IBD and pregnancy outcomes which can be cited to strengthen the background regarding inflammatory markers in IBD and pregnancy outcomes.

**Line 54 and 56:** Suggest to specify the adverse pregnancy outcomes for bacterial vaginosis and IBD. There are publications which mention that these are pre-term deliveries and low-birth weight deliveries. Also, your baseline data shows the same, so the mention of types of outcomes will support your introduction.

**Line 56-57:** The claim needs to be re-worded ‘They 56 are also at higher risk of poor pregnancy outcomes, yet their vaginal microbiota has not been described’. According to the ref: Hill JE, Peña-Sánchez JN, Fernando C, Freitas AC, Withana Gamage N, Fowler S. Composition and Stability of the Vaginal Microbiota of Pregnant Women With Inflammatory Bowel Disease. Inflamm Bowel Dis. 2022 Jun 3;28(6):905-911 – the microbiota is studied. It might not be studied widely, however the claim as written by you becomes untrue. In addition to that, addition of above ref will support your paper as it mentions that *L. crispatus* is the most commonly found microbe.

**Line 62-64:** This sentence is confusing and I would suggest to delete this line or find an alternative ref to support the claim. This line might be referenced to discussion part of Bar O et al (ref 12): “A few studies examining changes in vaginal microbiota over time in pregnant participants with IBD have not found significant differences from what has been described in pregnant people without IBD, aside from higher prevalence of *Mollicutes* in one cohort”. The Bar O et al shows that there is no association between IBD severity over time with changes in vaginal microbiome between pre and post menopausal women.

**Materials and Methods:**

**Line 75-84:** Recommend to describe MELODY trial briefly – for e.g. the MELODY (Modulating Early Life Microbiome through Dietary Intervention in Pregnancy) trial tests whether the IBD-AID™ dietary intervention during the last trimester of pregnancy can beneficially shift the microbiome of ……” from the relevant publication (Peter et al). I had to visit the publication to understand the original study and its inclusion/exclusion criteria.

**Line 80:** “Written consent”. Word consent is missing.

**Line 127-129:** Data imputation - use of median values- for missing data is not recommended. Ideally data from missing samples should be excluded or the last available data for the patient should be used. Recommend to do the analysis with excluding the patient values.

**Line 131:** spelling error “later”

**Results:**

**Line 202 to 206:** based on the title of the paper, I was expecting this to be first results which would be presented visually. I recommend to present this in tabular form. In addition to that, table S4 should be presented after figure 1B. Even though the results are not significant, it is important to demonstrate visually that the CST based microbiota diversity. In the table you can put a column saying P values NS. This would really strengthen the paper in terms of visually demonstrating that the authors have evaluated the data by subgroups and arrived at the conclusion which is presented in the paper.

**After line 228:** IL-6 results and its implication in disease is covered. However, equally interesting to note that IL-4 and IFN-g were lower. Higher IFN-g is associated with poorer pregnancy outcomes, so lower IFN-g levels in this study are interesting as the baseline characteristics show that there were 4 preterm births in IBD cohort. Exploring this would provide interesting insights.

**Line 282:** Do you mean fig 4? Table S4 is not showing diet associated data. Please verify.

**Table S6:** This data was very interesting to me. However, it is not described in the results section. HEI, total vegetable, total fruit and added sugar – all show significant association of variable to CST type I especially *L. crispatus* which is also mentioned in the abstract. These results should be described in the paper in a short paragraph.

**General editorial:** *Lactobacillus* is expanded at few instances and is shortened to L at few instances. Keeping it consistent would improve the editorial quality. (e.g., line 272 and 279)

**Discussion:**

**Line 324 and 325:** The authros demonstrate high fecal calprotectin (fig 1A). This well studied indicator of gut inflammation in IBD, so this needs to be mentioned in the sentence as it will improve your argument of high gut inflammation and corelate it to immune tone of vaginal environment.

**Line 331-333:** The role of IFN-g is not very well explored in this paper even though it was found to be lower in the study and showed association with diet. The authors state that lower IFN-g is associated with poor pregnancy outcomes, but I came across articles which stated that high IFN-g is associated with poorer outcomes. (ref: Jijon H, Ueno A, Sharifi N, Leung Y, Ghosh S, Seow CH. Elevated interferon-gamma levels during pregnancy are associated with adverse maternofetal outcomes in IBD. Gut. 2020 Oct;69(10):1895-1897 AND Hossein-Javaheri N, Youssef M, Jeyakumar Y, Huang V, Tandon P. The Management of Inflammatory Bowel Disease during Reproductive Years: An Updated Narrative Review. *Reproductive Medicine*. 2023; 4(3):180-197.) I am giving 2 examples here, however I suggest that authors should review the literature thoroughly to understand the role of IFN-g in pregnancy outcomes for IBD patients and thereafter elaborate on the IFN-g role in the discussion section. If this is a contradictory result, then it needs to be highlighted as a unique finding of this study.

**General observation:** The limitations of the study are well covered.
