# Supplementary material for: Association of vaginal IL-4, IL-6, IL-8, IL-17, IFN-γ, and dietary intake with IBD status and vaginal microbiota in pregnant individuals
Source: PLoS One. 2026 Jan 14;21(1):e0335178. doi: 10.1371/journal.pone.0335178 (PMC12803450; doi:10.1371/journal.pone.0335178)
Supplement: Attachment — Submitted filename: 20250922_Response to reviewers.docx [file pone.0335178.s014.docx]

**Manuscript: Association of Inflammatory Bowel Disease and Diet on IL-4, IL-6, IL-8, IL-17, and IFN-γ Vaginal Cytokines and Microbiota During Pregnancy**

**Round 2 Comments:**

**Overall comments:** The changes have greatly improved the quality of the Manuscript and I would like to thank the authors for incorporating comments. In this draft, I have provided direct suggestions for to expediate the process of re-submission.

In addition to the edits, during review I noticed few minor inaccuracies which were present in draft 1 as well as this draft. Incorporating all the comments will greatly enhance the quality of manuscript.

*We sincerely appreciate the reviewers’ time, as well as their thoughtful feedback and suggestions. We agree with all of the comments/suggestions. Our responses to each are provided below (and changes have been made throughout the manuscript) and highlighted in gray.*

**Specific comments:**

Line 1-2: the re-written title is now grammatically incorrect. Association is always “between/with”; it’s not “on”. Please use either or whatever you prefer to give a clearer meaning: **Association of Inflammatory Bowel Disease and Diet with IL-4, IL-6, IL-8, IL-17, and IFN-γ Cytokines and Microbiota in Vaginal Mucosa During Pregnancy**

**OR**

**Association of vaginal inflammatory markers such as Il-4, IL-6, IL-8, IL-17 and IFN-g and diet with IBD and vaginal microbiota in pregnant individuals**

*R: The manuscript is now entitled: “Association of Vaginal IL-4, IL-6, IL-8, IL-17, IFN-γ, and Dietary Intake with IBD Status and Vaginal Microbiota in Pregnant Individuals”*

Line 38 – line “Most of the IBD cases were in remission (56.5%)”. It should be A little over half OR mention that 56.5% cases were in remission. 56% would not be considered as “most” .

*R: The suggested change had been made.*

Line 40 – change cases to individuals.

*R: The suggested change had been made.*

Line 46 – word respectively can be removed.

*R: The suggested change had been made.*

Line 47: “Vaginal microbiome was not associated with IBD status in this cohort” - not able to understand this sentence. – which cohort? What part of vaginal microbiome was not associated?

*R: Changed to: “The vaginal microbiome did not differ between individuals with IBD and HC”*

Line 49-50: “Regardless of IBD status, healthier diets are positively associated with an increased abundance of the beneficial L. crispatus in the vagina”. Results section of abstract does not mention any results to support this line (the part which says regardless of IBD status) in conclusion.

*R: “regardless of IBD status” is now included in the results section.*

**Introduction:** Line 72: change diet to dietary

*R: The suggested change had been made.*

**Methods:** Line 130: “as we have previously done” – do you mean “previously mentioned”?

*R: Changed to: “as we have previously done in other studies” and we include the references to those published studies.*

Line 133: “as we previously described” – grammatically incorrect. In this sentence it should be “as described previously”.

*R: Changed to: “as described previously by us”*

Line 134: “diet quality used to assess 134 how well 13 groups of foods align with key recommendations” – grammatically incorrect. Should be “diet quality used to assess alignment of 13 food groups with key recommendations…”

*R: The suggested change had been made.*

Line 169-172: grammatical errors in sentence formation. Should be “Microbial taxa (at the species level) and their association with clinical 170 variables were assessed using MaAsLin2’s [40]. **Assessment was conducted only for the variables that were found to be** significant or marginally significant in the beta diversity analyses **and included** only the taxa **that was** present in at least 20% of the samples.”

*R: The suggested change had been made.*

**Results:** Table 1: why are p values presented? P values are used to test a hypothesis. In comparing baseline data the idea is that groups are comparable. Do the authors aim to show non-significant differences between the group? In that case you can mention that all groups were comparable and variables did not have significant differences. However, I request authors to justify the reason for using P values before accepting my suggestion.

*R: Thank you for your thoughtful comment regarding the inclusion of p-values in Table 1. In our study, the baseline characteristics of the groups showed no significant differences. P-values were included to objectively demonstrate comparability between the groups and to provide transparency in reporting, following common practice in clinical research. However, since all variables were comparable and there were no statistically significant differences, we can remove the p-values and instead note in the manuscript that the groups were similar across all measured baseline characteristics. We appreciate your feedback and are happy to revise Table 1 according to your recommendation.*

Line 213: I understand that this study is part of larger MELODY group, and hence individuals in this study are also technically a “cohort of MELODY”. However, for this publication we are talking about 48 individuals and cohorts are IBD and HC – so cohort becomes misleading, it might be better to use the term “study”.

*R: The suggested change had been made.*

Line 217: grammatical error – “not differences” to be changed to “no differences”

*R: The suggested change had been made.*

Lines 220-221: **MAJOR COMMENT:** in previous draft the TNF-a results were not presented and IL-6 results were segregated by Simpson and Shannon analysis. In this draft, TNF-a results are newly introduced. IL-6 results are non-significant for Shannon analysis and significant only for Simpson analysis. Please specify this clearly. The way it is worded is misleading.

*R: In this revised manuscript, we present results from a re-analysis of our data as requested by Reviewer #2:* *Amruta A Jambekar. Specifically, the reviewer recommended that we exclude patients with missing data that we had previously imputed with their median (2 for BMI and 4 for calprotectin levels), from our analyses. In the current version, we have excluded these patients only from the analysis that accounted for these two variables (BMI and calprotectin). This adjustment resulted in minor changes to our findings: TNF-α is now significantly associated with microbial diversity, whereas the correlation between IL‑6 and the Shannon index is no longer significant. Instead, IL‑6 now shows a significant correlation with the Simpson index.*

*That being said, we appreciate your careful review and for identifying the misleading language! Results are now described as follows: “Expression of IL-6 was positively associated with vaginal microbial diversity as measured by the Simpson index, but not by the Shannon index (****Fig. 2****). In contrast, TNF-α expression was significantly associated with vaginal microbial diversity according to both the Shannon and Simpson indices, regardless of IBD status (P < 0.050,* ***Fig. 2****).”*

Also, I am not sure why the p-values in draft 1 and R1 are different for IL-6, figure 2 (draft 1 0.032 and 0.126 for Shannon and Simpson respectively versus ns and 0.47 for Shannon and Simpson in R1). Please Justify.

*R: See answer above.*

Line 221-224: can this be presented after line 218? Suggest to write in same order as presented in figure 1.

*R: The suggested change had been made.*

Line 236: include TNF-a. i.e. higher IL-6 and TNF-a are associated with….

*R: The suggested change had been made.*

Line 254 and Line 256: **MAJOR COMMENT:** Figures show data and p values for Simson and Shannon analysis separately, but in line 254 it is presented as combined? P is ns for Simpson for added sugar intake, and 0.31 for Shannon diversity index. Please specific – current way of writing is misleading.

*R: Thank you for bringing this oversight to our attention. The suggested change had been made.*

Line 294: Again, the word study might be more suitable instead of cohort.

*R: The suggested change had been made.*

Line 239 and 299: **MAJOR COMMENT:** titles are incorrect. It is vaginal microbiota associated with diet or vaginal cytokines associated with diet. I understand that the authors want to highlight Association of diet with result outcomes – so suggest to use the titles: “Association of diet with……”

*R: The suggested change had been made.*

Line 308: were negatively associated “with” expression of

*R: The suggested change had been made.*

Line 309: word respectively can be removed

*R: The suggested change had been made.*

**Discussion:** Line 332: replace word most with “more than half or 56.5%”

*R: The suggested change had been made.*

Enjoyed reading the discussion, especially the limitations paragraph.

*We are grateful for your attention to detail and for pointing out mistakes, misleading writing, and grammar errors!*
